# Epigenetic control of ion channel expression and cell-specific splicing in nociceptors: Chronic pain mechanisms and potential therapeutic targets

**DOI:** 10.1080/19336950.2020.1860383

**Published:** 2021-01-08

**Authors:** Diane Lipscombe, E. Javier Lopez-Soto

**Affiliations:** The Robert J and Nancy D Carney Institute for Brain Science & Department of Neuroscience, Brown University, Providence, RI, USA

**Keywords:** Chronic pain, neuropathic pain, aberrant alternative splicing, epigenetic control, cell-specific splicing, morphine efficacy, Trpv1, DNA methylation, exon methylation

## Abstract

Ion channels underlie all forms for electrical signaling including the transmission of information about harmful events. Voltage-gated calcium ion channels have dual function, they support electrical signaling as well as intracellular calcium signaling through excitation-dependent calcium entry across the plasma membrane. Mechanisms that regulate ion channel forms and actions are essential for myriad cell functions and these are targeted by drugs and therapeutics. When disrupted, the cellular mechanisms that control ion channel activity can contribute to disease pathophysiology. For example, alternative pre-mRNA splicing is a major step in defining the precise composition of the transcriptome across different cell types from early cellular differentiation to programmed apoptosis. An estimated 30% of disease-causing mutations are associated with altered alternative splicing, and mis-splicing is a feature of numerous highly prevalent diseases including neurodegenerative, cancer, and chronic pain. Here we discuss the important role of epigenetic regulation of gene expression and cell-specific alternative splicing of calcium ion channels in nociceptors, with emphasis on how these processes are disrupted in chronic pain, the potential therapeutic benefit of correcting or compensating for aberrant ion channel splicing in chronic pain.

## Epigenetic reprogramming in neurological diseases and chronic pain

Pathological pain disorders are a growing healthcare challenge; in 2016, approximately 20% of adults in the United States alone live with a form of chronic pain [1]. The majority of FDA-approved chronic pain treatments are not uniformly effective across patients, the therapeutic effectiveness of current treatments within responsive cohorts is variable, and opioids and related analgesics are addictive and subject to tolerance during treatment. New therapies have been developed to target specific types of chronic pain that have well-described origins. For example, the use of CGRP blocking antibodies to mitigate pain associated with migraine [2,3]. But for most forms of chronic pain non-addictive highly efficacious treatments are lacking and opioids, including morphine, continue to have widespread use in the treatment of neuropathic pain despite the high doses required for analgesia [4].

A common feature of chronic pain is long lasting, pathological changes in neuronal excitability, originating from disease-dependent, cell-type specific alterations in the expression and function of ion channels [5–7]. Identifying cell-specific processes that control ion channel gene expression is critical if we are to understand more fully how neuronal injury leads to long lasting dysregulation of ion channel expression and function. Dynamic changes in epigenetic marks in different neuron subtypes, during development, and in response to changes in activity and environment coordinate expression levels of functionally related genes, including those that encode ion channels [8–10]. But, persistent reprogramming of the epigenetic landscape can lead to abnormal changes in gene expression and this is a common feature of many neurological disorders including Alzheimer’s and Parkinson’s diseases [11,12] Huntington’s Disease, Amyotrophic Lateral Sclerosis [13], Autism Spectrum Disorders [14], Rett Syndrome [15] and chronic pain [16]. In most, if not all of these diseases, prolonged alteration in epigenetic regulation impacts a large number of genomic loci which, as a consequence, results in chronically abnormal expression and actions of gene networks. There is strong interest in therapeutic approaches to reverse disease-associated changes in ion channel expression. Epigenetic-based therapies have proven effective in certain cancers and other diseases [17,18].

### Epigenetic signatures altered in chronic pain

DNA methylation, histone modifications and chromatin remodeling are interconnected processes that depend on a complex set of factors that combine to form the epigenetic machinery. Epigenetic factors influence the complete range of molecular interactions that control gene expression and these in turn influence transcriptome compositions of different cell subtypes, at different stages of development, following changes in activity and more. It is increasingly evident that a reprogramming of the epigenetic landscape (DNA, histones, and chromatin) in affected cells is a feature of many diseases, including in neuropathic pain [16,19–21]. Moreover, epigenetic marks can be used to inform disease origin and treatment strategies [17,22].

In neuropathic pain, injured neurons exhibit altered histone modification patterns that impact protein access to gene loci [16,21,23] including transcription factor occupancy at gene promotor regions [21,24], and altered DNA methylation of gene promoters and alternatively spliced exons [19,25–27].

*Histone modifications*, principally acetylation, methylation, and phosphorylation, are essential elements involved in the dynamic control chromatin structure. These histone modifications are key for regulated, coordinated gene expression and RNA processing. For example, in affected dorsal root ganglia (DRG) after nerve injury, there is increased methylation of lysine 9 of the histone H3 complex (H3K9) via upregulation of the lysine methyltransferase G9a. Injury-induced accumulation of dimethylated H3K9 (H3K9me2) in gene promoter regions represses transcriptional activity of more than 600 genes [16,23]. Among these genes, *Kcna4, Kcna2, Kcnd2*, and *Kcnq2* encode four different voltage-gated potassium ion channels that collectively function to curtail neuronal excitability. H3K9me2 within the promotor regions of these genes down regulates K_V_ channel expression in affected DRG causing increased neuronal excitability and contributing to neuropathic pain pathology [16,23,28]. Such coordinated action over several functionally related gene loci illustrates the hierarchical position that epigenetic mechanisms exert in the dynamic control of neuronal excitability.

*DNA methylation* occurs primarily on cytosines followed by guanines (CpG) and specifically on the 5th carbon of the pyrimidine ring of cytosine (5-methylcytosine or 5mC). CpG methylation is catalyzed by DNA methyltransferases (DNMTs) of which DNMT3a and DNMT3b promote *de novo* methylation of CpG sites involved in dynamic control of gene expression. DNA demethylation oxidation is catalyzed by Ten Eleven Translocation (TET) enzymes (TET1, TET2, and TET3). TETs convert 5mC into 5-hydroxymethylcytosine (5hmC) [29]. A comparison of CpG methylation patterns in unaffected and affected DRG in peripheral nerve injury models shows alterations in ~15% of methylation loci [19]. CpG methylation across the genome is overall reduced across the genome of affected DRG neurons [19,27]. Dynamic CpG methylation regulates neuronal intrinsic membrane excitability and it has been implicated in neuropathic pain [30]. Therapeutic strategies that increase CpG methylation might be expected to alleviate or reverse some of pathophysiological changes associated with peripheral nerve injury. Interestingly, methyl donor-enriched diets have been reported to attenuate certain sensory pathologies associated with nerve injury in mice [31,32], although the mechanisms of action of methyl donor enriched diets remain to be established.

The majority of nerve injury-induced alterations in genome-wide CpG methylation patterns are located in intergenic regions (between genes) [19] which contain regulatory mechanisms that control gene expression. CpG methylation at the promoter region of the μ-opioid receptor gene, *Oprm1*, is increased in affected DRG following nerve injury. This hypermethylation of the *Oprm1* promoter impairs Oprm1 RNA processing leading to reduced levels of μ-opioid receptor expression [26,33]. Downregulation of μ-opioid receptors in injured neurons reduces neuronal responsiveness to opioid agonists and this likely contributes to the loss of morphine analgesia in neuropathic pain conditions [26,33]. As we discuss below, injury-induced alteration in cell-specific CpG methylation of the *Cacna1b* gene also results in reduced morphine efficacy but via changes in alternative splicing, not gene expression [34–36].

## Cell-specific alternative splicing of Ca_V_2.2 channels in heat-sensing nociceptors

**Figure 1.**
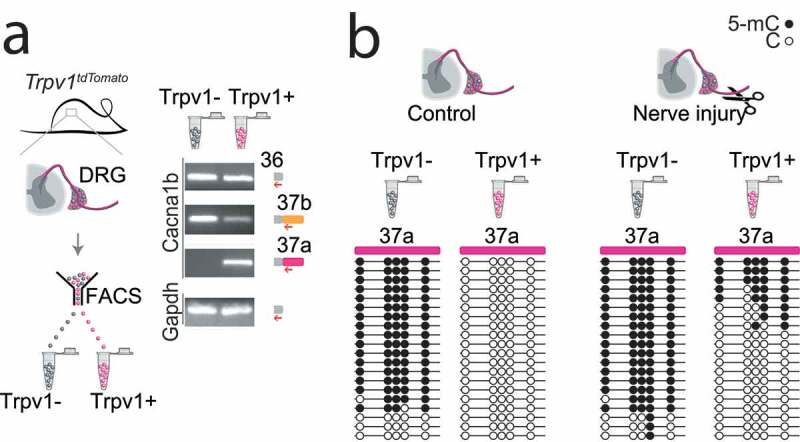
Cell-specific *Cacna1b* e37a inclusion and methylation in Trpv1 nociceptors is disrupted following nerve injury

**Figure 2.**
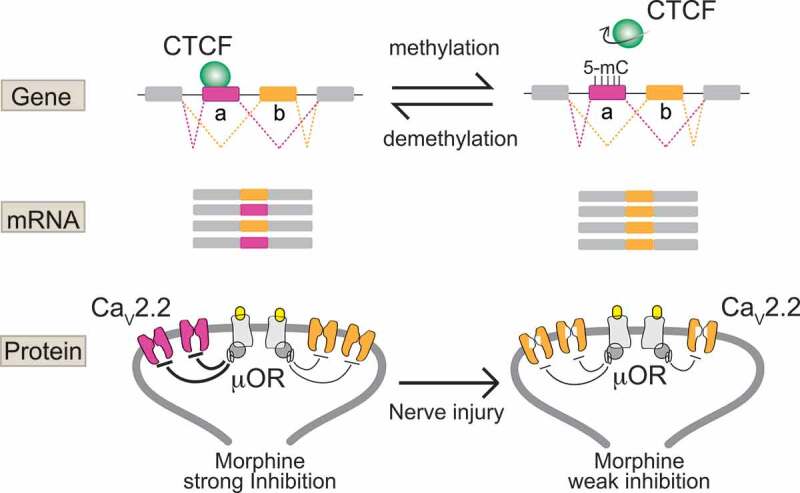
Cell-specific epigenetic factors control functional splicing in nociceptors

Ca_V_2.2 channels at presynaptic termini of nociceptors in the spinal cord dorsal horn regulate glutamate release and thus synaptic efficacy. As has been amply documented, Ca_V_2.2 channel inhibitors alleviate intractable chronic pain in humans, as well as in various animal models of pain [37–40]. Ziconotide (Prialt®), opioids and gabapentinoids downregulate Ca_V_2.2 channel activity in nociceptors by distinctly different mechanisms: occluding the ion pore of the Ca_V_2.2 channel directly (ziconotide); interfering with channel gating via G-protein coupled receptor activation (morphine); or acting via the α2δ-1 protein to promote Ca_V_2.2 channel internalization (gabapentin) [36,41]. Interestingly, the inhibitory efficacy of opioids on Ca_V_2.2 channels via μ-opioid receptor activation differs between e37 C-termini splice isoforms of Ca_V_2.2. As summarized in Figures 1 and 2, in heat-sensitive Trpv1 nociceptors, the e37a splice form of Ca_V_2.2 is expressed and it is more strongly inhibited by μ-opioid receptor activation especially under conditions of prolonged excitation. By comparison, the more commonly expressed splice form of Ca_V_2.2, e37b, dominates in most other neurons and it is less sensitive to μ-opioid receptor inhibition [25,35,42–46]. This difference in morphine efficacy, between Ca_V_2.2 splice isoforms impacts behavior. The intrathecal analgesic effects of morphine *in vivo* [35] and, most relevant to this discussion, abnormal cell-specific splicing of Ca_V_2.2 in heat-sensitive Trpv1 nociceptors following peripheral nerve injury, leads to reduced expression of the e37a splice isoform of Ca_V_2.2 and an accompanying reduction in the analgesic efficacy of morphine [25,34,36]. Disease-associated changes in alternative splicing of Ca_V_2.2 are likely to contribute to the reduced therapeutic efficacy of morphine in neuropathic pain [25,33,34,36,47–49]. Approaches to prevent or correct persistent changes in cell-specific alternative splicing of Ca_V_2.2, and potentially other synaptic proteins, in affected nociceptors could prove beneficial and might improve morphine efficacy in the treatment of chronic pain.

(a) *Trpv1^Cr^*^e^ mouse strain crossed to *lox-STOP-lox^TdTomato^* expressed the TdTomato reporter in Trpv1-lineage neurons (Trpv1^TdTomato^). DRG cells were separated into Trpv1-lineage and non-Trpv1-lineage cells by fluorescence-activated cell sorting (FACS). Using specific primer pairs RT-PCR products amplified: constitutive Cacna1b e36, alternative spliced e37a and e37b, and Gapdh in Trpv1-lineage and non Trpv1-lineage cells for all primer pairs except for non Trpv1-lineage cells that failed to amplify from e37a-specific primers. (b) Methylation in *Cacna1b* e37a locus increased and e37a inclusion decreased in Trpv1 neurons following peripheral nerve injury. Methylation levels of 5 CpG sites in *Cacna1b* e37a locus in non Trpv1- and in Trpv1+ neurons from L3 and L4 DRG ipsilateral to the site of surgery for sham (left, control) and peripheral nerve injury (right) mice. Methylated (5-mC, filled circle) and unmethylated (C, open circle) sites are shown for independent clones ordered from most to least methylated. Each set of sequences represents data pooled from L3 and L4 DRG from three animals. This figure is modified and reproduced from [25] in accordance with *eLife* CC-BY license.

Exons 37a and 37b are mutually exclusive in Cacna1b mRNA. Left, in Trpv1 nociceptors, the master chromatin regulator CTCF binds unmethylated e37a locus in *Cacna1b* gene and promotes e37a inclusion in mRNAs. Ca_V_2.2-e37a channels are strongly inhibited by μ-opioid receptor (μOR) activation, promoting morphine analgesia in spinal cord. DNMT3a promotes, and TET1/2 reduces, e37a methylation (5-mC) levels in *Cacna1b* respectively. Right, following nerve injury that results in neuropathic pain, e37a methylation (5-mC) levels increase, CTCF binding is impaired, and e37a is skipped and e37b is included in mRNAs. Ca_V_2.2-e37b channels are weakly inhibited by μOR activation. This disrupted splicing pattern following nerve injury is associated with a concomitant reduced efficacy of morphine *in vivo*. This figure is modified and reproduced from [25] in accordance with *eLife* CC-BY license.

## Epigenetic mechanisms underlie cell-specific alternative splicing of Ca_V_2.2 channels

Until recently, we knew virtually nothing of the factors that control cell-specific alternative splicing of ion channels in affected nociceptors in chronic pain models. This knowledge gap persists despite compelling and growing evidence for the critical importance of this process in normal and abnormal neuronal function [36,50–52]. This changed with our recent discovery that cell-specific epigenetic modification controls cell-specific alternative splicing of e37a in *Cacna1b* in Trpv1 sensitive nociceptors, and that nerve-injury triggers epigenetic reprogramming altering splicing and calcium ion channel function (Figures 1 and 2) [25]:

1. In most neurons and in non-Trpv1 DRG cells, five CpG sites in exon 37a of *Cacna1b* are strongly methylated and exon 37a methylation prevents binding of a CCCTC binding factor (CTCF) to its motif at this locus. CTCF is a ubiquitous DNA binding protein that has a wide range of functions [53]. By contrast, in Trpv1 nociceptors, the same five CpG sites in exon 37a of *Cacna1b* are unmethylated, or rarely methylated, allowing CTCF to bind to its motif in exon 37a of *Cacna1b*.
2. Exon 37a is defined by a weak splice junction; in most cells, the intron-exon 37a boundary is not recognized by the spliceosome, exon 37a is normally skipped during pre mRNA splicing, and the mutually exclusive exon 37b which is defined by a strong splice junction is included.
3. In Trpv1 nociceptors, CTCF binds exon 37a converting a weak slice junction to one that is now recognized by the spliceosome [54], and exon 37a is now approximately as likely to be included as exon 37b. Because exons 37a and 37b are mutually exclusive when exon 37a is included, e37b is excluded.
4. Days after peripheral nerve injury that leads to prolonged hyperalgesia, the hypomethylation state of exon 37a in Trpv1 nociceptors is disrupted; there is a persistent increase in exon 37a methylation, a decrease in exon 37a inclusion and as an accompanying increase in exon 37b inclusion in Trpv1 nociceptors ipsilateral to the injury.
5. The persistent disruption in the normal hypomethylation state of exon 37a in *Cacna1b* in Trpv1 nociceptors is coupled to reduced inhibitory actions of μ-opioid receptor activation on Ca_V_2.2 channels in affected neurons, and reduced intrathecal morphine analgesia in behavior.

Our findings raise a number of questions including is cell-specific, exon-specific CpG methylation a general mechanism controlling cell-specific alternative gene splicing of ion channels and other genes in nociceptors? Are DNA methyltransferases (DNMTs) normally restricted from e37a and do DNMTs gain access to this site after injury? Can abnormal CpG methylation of e37a be prevented or reversed to normalize certain aspects of chronic pain pathophysiology?

## Is cell-specific epigenetic modulation a general mechanism to control gene splicing?

Genome-wide epigenetic analyses in animal models of chronic pain reveal consistent, associated changed in DNA methylation and altered expression of hundreds of genes in affected DRG [19,27,55,56] and genome-wide CTCF mapping studies suggest a role for CTCF in control of splicing [54,57,58]. Such studies highlight DNA methylation as a common mechanism controlling the expression of gene networks according to changes in neuronal excitation [56,59]. Dynamic cell-specific changes in gene expression likely play important roles in normal homeostatic and synaptic plasticity that drive transient protective alterations in behavioral responses to stimuli. But, persistent, reprogramming of DNA methylation across neuronal genes in affected DRG post nerve injury, parallel disease pathology associated with chronic pain [19,20]. The proteins most commonly implicated in altered DNA methylation patterns associated with chronic pain are the DNA methyltransferases DNMT3a and DNMT3b [25,26,56,60].

DNMT3a is implicated in hypermethylation of key gene loci in affected DRG neurons following peripheral nerve injury, and in methylation of gene loci involved in calcium ion channel splicing in DRG nociceptors [25,26,56,60]. Specifically, in injured DRG, DNMT3a facilitates methylation levels at the *Oprm1* promoter region leading to gene silencing and, similarly at the promotor region of voltage-gated K_V_ channel *Kcna2* gene via activation of the transcription factor octamer transcription factor 1 [26]. In addition, as we have reported, overexpression of DNMT3a in a DRG-derived cell line increases the methylation state of the *Cacna1b* e37a locus suppressing e37a inclusion during alternative splicing (Figure 2) [25]. Thus, DNA methylation at specific loci (gene promotors or alternatively spliced exons) of functionally related genes in sensory neurons increases in response to nerve injury. The ensuing alterations in gene expression and RNA processing impact protein function and alter neuronal excitability and behavior relevant to pathological changes associated with peripheral nerve injury-induced chronic pain.

Our studies of Trpv1 nociceptor-specific hypomethylation of the e37a locus illustrate the importance of subtype-specific analyses of methylation modifications within specific genes (Figure 1) [25]. Indeed, the cell-specificity of CpG methylation of *Cacna1b* e37a locus would not be resolved in analysis of unsorted DRG. Thus, it will be important to define the genes whose expression and splicing patterns are modified by DNMT3a within genetically and functionally defined subtypes of sensory neurons. Deep sequencing analyses of different subtypes of DRG neurons show substantial differences in their gene expression profiles and, where available, of splice patterns [55,61,62].

## How does DNMT3a gain access to normally protected gene loci after nerve injury?

An as yet unresolved question of great importance is how, following nerve injury, DNMT3a promotes hypermethylation of normally hypomethylated regulatory loci in neuronal genes that contribute to disease pathology. The specificity of DNMT3a action at specific loci is not easily explained simply by an overall increase in DNMT3a levels, and it favors a model in which DNMT3a gains access to gene loci, that it is normally excluded from, in affected nociceptors after injury. Indeed, following nerve injury, previously protected regions of the chromatin become accessible to several DNA binding proteins [21]. In support of this hypothesis, DNMT3a levels are upregulated and DNMT3a accessibility to uniquely hypomethylated gene loci is increased in affected DRG for days in peripheral nerve injury models [25,26,56]. For example, promoter regions of neuronal genes, including *Oprm1, Oprk1, and Kcna2* become hypermethylated in affected, but not in unaffected DRG [26,56,63]. Resolving the mechanism that controls DNMT3a, and more broadly all DNMTs, accessibility will be an enormous step forward in elucidating the key factors that might be disrupted following injury and in disease.

In one of the few studies to address the critical question of how DNMT3a gains access to loci, normally not accessible, Miyanari and colleagues implicate promyelocytic leukemia (PML) bodies. PML nuclear bodies are membrane-less, spherical organelles ∼0.1–1 μm in diameter. They form 3-dimensional nuclear structures that physical exclude DNMT3a from specific gene loci to maintain hypomethylated regulatory elements crucial for the dynamic control of cell function [64]. It will be interesting to know whether the integrity of PML bodies is disrupted after nerve injury allowing access of DNMT3a to previously protected loci.

An alternative, not mutually exclusive possibility is the possibility that there is injury-induced reduction in the action of demethylating ten-eleven translocases (e.g. TET1 and TET2) at key loci. In our recent studies, we showed that localizing TET1 to the e37a locus in *Cacna1b* using dCas9 DNA targeting reduces CpG methylation and impacts e37a splicing in a DRG-neuroblastoma cell line [25]. While TETs and potentially other regulatory proteins could contribute to alterations in gene expression and splicing, active methylation by a DNMTs would seem to be necessary to explain persistent hypermethylation of key CpG sites following nerve injury [65–68].

## Can abnormal methylation be prevented or corrected to effectively normalize certain aspects of chronic pain pathophysiology?

Knowledge of cellular mechanisms that control gene expression should inform the development of effective therapies to treat chronic pain states. Therapies that reverse or normalize injury-induced epigenetic reprogramming in nociceptors, including CpG methylation, hold promise for normalizing protein activity and drug responsiveness in certain pain disorders. For example, preventing injury-induced DNMT3a CpG methylation of e37a in *Cacna1b* and at *Oprm1, Oprk1, and Kcna2* promotors in nociceptors could maintain normal levels of neuronal excitability and morphine efficacy.

Informed by careful mechanistic studies, therapeutic tools have been developed to compensate for damaging consequences of aberrant alternative splicing [69–72]. DNA engineering technologies, such as CRISPR-Cas9, have been employed to correct pathogenic epigenetic remodeling in neurons that interfere with alternative splicing, by compensatory shifts in alternative splicing, away from nonfunctional, toward functional splice isoforms [73–75]. As discussed above for *Cacna1b*, TET1 fused to CRISPER-dCas9 allows selective targeting of loci for *in vitro* and *in vivo* uses [25,76]. We applied this strategy to demethylate CpG sites in e37a, increasing e37a expression in a DRG-derived cell line [25] which suggests that this strategy could be used *in vivo* to counter hypermethylation following nerve injury.

As we gain knowledge of cell-specific epigenetic control of gene expression and splicing in sensory neurons, we come closer to developing new approaches to prevent and reserve epigenetic reprogramming associated with chronic pain pathology. Identifying cell-specific epigenetic control mechanisms, in particular, will be essential for achieving efficacy and specificity of drug action.

## Conclusion

Dynamic, cell-specific modulation of ion channel action through altered expression levels and altered alterative splicing is essential for neuronal plasticity, adaptation and homeostasis in neurons. Signature epigenetic reprogramming in subsets of neurons occurs during periods of excessive neuronal activity associated with nerve injury, environmental exposure to toxins, or ongoing chronic diseases. We have highlighted CpG DNA methylation as an example of injury-sensitive, nociceptor-specific epigenetic control of calcium ion channel splicing but we expect this mechanism will be found to regulate splicing of other functionally related ion channel genes. Knowledge of epigenetic mechanisms that regulate cell-specific ion channel gene expression and alternative splicing patterns will inform new therapeutic approaches to correct or to compensate for epigenetic reprogramming in chronic pain.
